# “I shut it out”: expectant mothers’ fear of childbirth after a traumatic birth—a phenomenological study

**DOI:** 10.1080/17482631.2022.2101209

**Published:** 2022-07-19

**Authors:** Barbo Olsen, Anikken Forgaard, Anne-Hedvig Salmi Nordsletta, Eva Sommerseth, Idun Røseth

**Affiliations:** a Centre for Women, Family and Children’s Health, Faculty of Health and Social Sciences, University of South-Eastern Norway, Kongsberg, Norway; b Department of Child and Adolescent Mental Health, Skien, Norway

**Keywords:** Fear of childbirth, birth anxiety, traumatic birth, pregnant women, expectant mothers, midwife, phenomenology, experience

## Abstract

**Purpose:**

To describe expectant mothers’ experiences of fear of childbirth after a previous traumatic birth.

**Method:**

Qualitative, individual, in-depth interviews were conducted with eight expectant mothers between September and November 2020. Data were analysed using a descriptive phenomenological approach.

**Results:**

We identified four interconnected constituents: Suboptimal midwifery care, loss of control and agency; insufficient time and capacity to process a traumatic birth experience; “The baby has to be delivered!”, and finally, the path to a new childbirth with the hope of mastering fears.

**Conclusion:**

Findings reveal an association between a previous history of traumatic birth and a fear of childbirth in expectant mothers. The trauma they experienced whilst giving birth strongly impacted their subsequent pregnancy, making it difficult for them to deal with the new pregnancy and impending birth. Women who have experienced a traumatic birth need to have the opportunity to process the trauma. Routines must therefore be developed that identify, support and follow up with the women. If these women are identified and given the help they need, this may help prevent and/or alleviate fear of childbirth in their subsequent pregnancy.

## Introduction

Childbirth is a significant event in a woman’s life. The act of giving birth often represents the most intimate and personal experience a woman will have. Giving birth is thus not only a medical event, but also a psychological, existential and social event (Blåka, 2002). Anxiousness before an impending birth is normal for expectant mothers; however, some women’s anxiousness will develop into a fear of childbirth—or even a severe fear of childbirth, known as tokophobia (Henriksen et al., 2020; Wigert et al., 2020).

Recent international prevalence studies estimate that the prevalence of tokophobia is almost 14%, with significant differences across countries. In Norway, the prevalence of tokophobia is around 7% (Lukasse et al., 2014; Nilsson et al., 2018; O’Connell et al., 2017). As the term “fear of childbirth” is ambiguous and has no standard definition (O’Connell et al., 2017), when an expectant mother expresses a fear of childbirth and seeks help, this in itself should be viewed as a valid definition (Saisto & Halmesmäki, 2003). The Norwegian health services’ (Helsenorge, 2020) website describes fear of childbirth as severe anxiety or a fear of giving birth, where the anxiety can be so overwhelming that it significantly impacts everyday life.

Severe fear of childbirth is further categorized as primary tokophobia in nulliparous women and secondary tokophobia in multiparous women. The reasons for which a woman might fear childbirth are complex, and can vary between nulliparous and multiparous women. Primary tokophobia can stem from adolescence (Hofberg & Brockington, 2000), and studies have shown that fear of childbirth in nulliparous women can be the result of childhood abuse (Lukasse et al., 2010). Secondary tokophobia is often related to a fear that has developed following a traumatic birth (Hofberg & Brockington, 2000), and a previous traumatic birth is a strong predictor of fear of childbirth in multiparous women (Dencker et al., 2019; Eide et al., 2019; Lukasse et al., 2010; Størksen et al., 2013).

A traumatic birth can lead to a diverse set of negative outcomes, including disrupting a woman’s sense of identity (Thomson & Downe, 2010), enduring mental health problems (Beck, 2015; Berg & Lundgren, 2004; Ertan et al., 2021), and maternal–child relationship difficulties (Nicholls & Ayers, 2007); it can also impact women’s reproductive decisions (Fenech & Thomson, 2014; Gottvall & Waldenström, 2002). A birth can be described as traumatic if a woman experiences a strong sense of anxiety, helplessness, loss of control, powerlessness or fear (Beck, 2015). Women’s subjective birth experience has been shown to be the main contributing factor for developing post-traumatic stress symptoms after childbirth (Garthus-Niegel et al., 2013). Moreover, women who have experienced a traumatic birth may find that a new pregnancy triggers negative memories and a fear that history will repeat itself (Sheen & Slade, 2018).

There is a growing body of research on traumatic birth and its consequences (Anderson, 2017; Beck, 2015; Beck & Watson, 2010) and on fear of childbirth (Wigert et al., 2020). However, few studies have focused specifically on women’s fear of childbirth after a traumatic birth. More research is needed on women’s experiences of fear of childbirth to gain a better understanding of the phenomenon. This could help ensure that women receive the best possible support and treatment in clinical practice, as well as illuminate factors that could reduce the incidence of fear of childbirth (Hildingsson et al., 2017; Klabbers et al., 2016; O’Connell et al., 2017). Understanding how and why some women’s fear of childbirth increases with subsequent pregnancies is an important element in the development of future maternity care (Eide et al., 2019). The purpose of this study was to describe expectant mothers’ experiences of the phenomenon of fear of childbirth after a previous traumatic birth. Throughout the article, the term “healthcare professional” will be used; this mainly refers to midwives and obstetricians.

## Method

We chose to employ a phenomenological method for this study. Phenomenology is based on the lifeworld—the concrete reality in which the world is experienced and lived (Giorgi, 2009; Merleau-Ponty, 2012). Husserl is considered to be the founder of modern phenomenological philosophy, which was developed to study the inner world and consciousness of humans and how things appear in our consciousness, namely phenomena. Consciousness is characterized by intentionality, which means that it is always directed towards something other than itself (Merleau-Ponty, 2012).

Adopting a disciplined form of empathy is an important principle of the method. This entails an attitude shift, which Husserl refers to as “*epochè*”, also known as “bracketing” (Giorgi, 2009; Morley, 2010). In this approach, the researcher tries to rid himself of prejudice and knowledge about the subject under investigation, and directs his empathic attention towards the experiences of the other. This is necessary in order to see the phenomenon in a new light: the researcher projects himself into the other’s point of view and sees the world through “their eyes”. Phenomenological psychological reduction is a continuation of the *epochè* approach; here, the researcher refrains from making a judgement on whether or not something exists in reality, and only concerns himself with what is presented to the consciousness—namely, phenomena (Giorgi, 2009).

### Participants

A purposive sample was employed in order to shed as much light as possible on the phenomenon. Midwife colleagues at counselling centres affiliated with two hospitals in Norway selected and recruited eight participants into the study. These midwives had no further involvement in the research process. The inclusion criteria were expectant mothers with a previous traumatic birth experience and a severe fear or anxiety of childbirth. The birth traumas were self-defined and not dependent on any objective measures. Expectant mothers were considered as having a fear of childbirth if they expressed a fear of giving birth and sought help from professional healthcare personnel. First-time mothers were not included, nor were women who did not speak Norwegian (to avoid language barriers).

Ten eligible women were invited to participate in the study, of which eight gave their informed consent. The women ranged from 22 to 39 years of age. Seven of the participants had completed higher education. They were all married or cohabiting and all reported that the pregnancy was planned. All the women had previously experienced a traumatic birth. The period between the traumatic birth and the new pregnancy ranged from 2 to 16 years. In the case of previous deliveries, six women had a normal vaginal birth and two had a caesarean section. The women were all multiparous; one was expecting her third child, while the remaining eight had one child and were giving birth for the second time. The gestational age of the new pregnancy at the time of the interview varied from week 20 to 35. All the women were receiving aftercare from a midwife at the counselling centre at the hospital where they planned to give birth. They had been referred by the specialist health service, a community midwife or a general practitioner, due to their need for follow up outside the primary care services.

### Data collection and analysis

We conducted individual in-depth interviews from September to November 2020. We interviewed the participants face-to-face at the outpatient clinic, where the surroundings were familiar to them. All interviews lasted between 40 and 65 minutes. We developed an interview guide with open-ended and flexible questions, which we tested via two pilot interviews. The interviews commenced with the open-ended question “Do you want to talk about how you experienced your previous childbirth?”, to encourage participants to talk about their experiences from previous childbirths. They were then asked to describe their experiences from when they discovered they were once again pregnant to the present day: “Feel free to talk about the process from when you became pregnant until now—how has it been for you?” Questions were then posed to elicit their thoughts and expectations in relation to the impending birth: for example, “What thoughts do you have about the time leading up to your impending birth?” The final questions concerned their perspectives on what would be important during the birth and what would constitute a high standard of aftercare: “What are your expectations for this birth? What will be important to you during this birth, and what might be the best follow-up care?” Subordinate questions centred on their experience of the new pregnancy and dealing with everyday life, and their perceptions of their relationship with their loved ones. The interviews were audio recorded and transcribed verbatim.

A descriptive phenomenological method was employed to analyse the data on participants’ fear of childbirth after a previous traumatic birth. This method is suitable for a phenomenological study and is closely linked to Husserl’s phenomenological philosophy. As recommended by Giorgi (2009), the analysis was carried out in a four-step process, where the first step was to read the transcribed interviews several times to form an overall impression. In the second step, we divided the text into meaning units to reflect different meanings, whilst continuing to focus on the phenomenon. Giorgi (2009) refers to the third step as “the heart of the method”; here, the meaning units are transformed from natural expressions into phenomenological psychologically sensitive expressions. The aim is to uncover and extract meanings from the descriptive narratives, and the researcher adopts an attitude of phenomenological reduction and uses his intuition when processing the material. We used the “imaginative variation” method to identify the most precise descriptions of the meanings in each unit, with sensitivity towards the phenomenon being investigated (Giorgi, 2009). As Merleau-Ponty (2012) argues, returning to the thing itself is key, as is finding its essence; this process is called “eidetic reduction”, and is an important phenomenological principle (Giorgi, 2009). In the fourth step, we synthesized the transformed meaning units into a common general meaning structure.

In our analysis, we did not follow a strictly linear process; rather, it was a dynamic process involving movement back and forth between the various steps. When analysing the interviews, we critically examined and discussed the delineation and meaning of the different meaning units. We detected some initial differences with respect to the identified meaning in the text, which were resolved upon further reflection and discussion.

### Reflexivity

The research team’s background, clinical experience and theoretical knowledge of birth anxiety can be viewed as a strength as well as a limitation when conducting a study on women’s lived experiences of the phenomenon. All authors in this study have a background as health professionals; four are midwives and one is a clinical psychologist. All have clinical practice caring for women and their families during pregnancy, birth and the postnatal period.

We recognized that, as researchers, our preconceptions about birth anxiety may inadvertently affect the research and presentation of findings. Our clinical experience was likely what initially sparked our interest and motivated us to conduct research on the topic. To ensure openness to the phenomenon, we engaged in a phenomenological approach involving *epoché* and phenomenological reduction, as described above. Through a disciplined, empathetic attitude (*epoché*), we sought the subjective experiences of the participants. By setting aside our own prejudices and asking in-depth questions, we opened up to the other’s experience of their lifeworld (i.e., how the women experienced and described their birth-related anxiety). To further ensure the validity of our findings, we conducted a systematic, transparent and descriptive analysis of the data.

### Strengths and limitations

We used in-depth interviews and a descriptive phenomenological method to analyse the data; this approach proved well-suited to illuminating the women’s experiences, as it allowed them to talk freely and openly about the phenomenon of fear of childbirth after traumatic labour (Giorgi, 2009). One limitation is that our findings are not statistically generalizable to the whole population, as our study was qualitative, with a small, non-representative sample. Qualitative findings, however, may be transferable to other women in similar situations, and generalizable to the phenomenon under study. We interviewed eight Norwegian women over a relatively short period, and acknowledge that women from other socio-cultural contexts may have different experiences. At the same time, we also avoided any language barriers that could lead to misinterpretation of the data.

We focused on the meaning of the women’s subjective experience, and did not include objective measures of birth trauma or fear of childbirth. While this may be seen as a limitation, in a phenomenological study, it is important to let the participants define these phenomena qua their experience, rather than rely on standardized criteria or theoretical construct-based questions. The eight women who responded to the invitation wanted to share their experiences and contributed with rich descriptions, providing sufficient information to illuminate the phenomenon (Malterud, 2017). Further strengths include the fact that we conducted the interviews face-to face, and that the setting was the same for all the participants; moreover, the interview guide contributed to a consistent interview structure and process.

The fact that all the women were pregnant at the time of the interview allowed us to obtain rich descriptions of their present experience of fear of childbirth, rather than relying on their recollections. For some participants, however, several years had passed since the traumatic birth, so they may not have remembered all the details; this represents a limitation. In addition, we did not include information about other elements that may have affected the women’s experiences, including their partner’s role and attendance at antenatal classes.

### Ethics

The study was conducted in accordance with the Declaration of Helsinki (World Medical Association, 2013), and was approved by the Regional Committees for Medical and Health Research Ethics (REK: 143,504) and the Norwegian Centre for Research Data (NSD: 140,686). The participants received both written and oral information about the study, and were informed that participation was voluntary and that they could withdraw from the study at any time without giving a reason. They agreed to participate by signing the consent form before the interviews began. The participants were given the opportunity to talk to a midwife after the interview if they so wished, and one participant accepted this offer.

## Results

In the interviews, the women shared their experiences of fear of childbirth after a previous traumatic birth. We identified a general meaning structure within these experiences, as detailed below.

For the women in our study, fear of childbirth after a traumatic birth was experienced as a strong feeling of insecurity, with a fear that history will repeat itself. This fear accompanied their new pregnancy, as they feel forced to relate to the idea of giving birth again. It also became a barrier to feeling happy about the pregnancy and preparing for the birth and the postnatal period (both mentally and practically). The women described their previous traumatic birth as an experience of heightened insecurity and loss of control. Their childbirth experience differed from their expectations, and they were unprepared for how painful and difficult it turned out to be. They linked the loss of control they experienced to inadequate or unclear information and absent midwives, making them feel alone. The participants also described a loss of agency, and felt that they were not seen or heard, nor were they included in the decision making. For some, this led to difficulties after the birth, in terms of having the emotional availability to feel warmth and love for their child; they also experienced a reduced capacity to actively participate in their role as a mother. This in turn evoked the feeling that they were not coping. The women felt that they had limited capacity to process the traumatic birth, and their coping strategy was to suppress the trauma and put it behind them. This strategy had severe implications for the women’s subsequent pregnancy, with flashbacks and a fear that their experience would be repeated. They struggled to deal with the traumatic birth, new pregnancy and impending birth. The participants also expressed ambivalence: they hoped that the birth would go well, while simultaneously feeling great uncertainty and fear that it would not. They called for autonomy and a wish to be heard, seen and included in their own birthing process.

We identified four highly interrelated essential constituents in the general meaning structure that we have separated for the sake of analysis: Suboptimal midwifery care, loss of control and agency; insufficient time and capacity to process a traumatic birth experience; “The baby has to be delivered!”, and finally, the path to a new childbirth with the hope of mastering fears. These four constituents are described in more detail below. We present the constituents in chronological order, while illuminating their interconnectedness and relevance for the women’s experience of fear of childbirth.

### Suboptimal midwifery care, and loss of control and agency

The women in our study had previously had a traumatic birth experience that evoked overwhelming memories when they were faced with a new pregnancy. The traumatic birth experience involved intense pain, a loss of control, insecurity, a fear of death and uncertainty about surviving the birth. They also expressed a desire to flee the situation. Contributing to the traumatic birth experience, they described periods with suboptimal midwifery care, in which they felt alone or like they were not treated with respect. Childbirth was not what they had anticipated, and they were unprepared for shortcomings in their care or dramatic complications that arose during the birth.

It emerged that a lack of information contributed to feelings of insecurity and anxiety, and the women reported having received insufficiently clear communication and guidance. They described the trauma of their first birth as a sudden shift, from feeling as though they were coping to feeling a loss of control, as well as helplessness and insecurity.

*I actually felt a bit like I was being ignored when they wouldn’t tell me anything about what was going on, I was just kind of told that a gynaecologist was going to try suction and forceps. Then more of them just kept appearing. It was like all information was being withheld at the point when I was so in need of reassurance*. (w4)

The way the information was transmitted was important to the women. To be prepared, they wanted to be given proper information prior to any interventions. The women explained that their insecurity and anxiety was triggered when suddenly, without warning, various people appeared in the delivery room (as seen in the above quote). One participant described this as follows: “*I woke up to a commotion; a doctor and several other people had entered the room. They didn’t talk to us, they just looked at the screen because at that point the baby’s heart rate had dropped quite a bit*” (w3).

Several women reflected on their exclusion from the decision making surrounding their birth. They did not feel included in the decision being made about them, and found that it was the midwife who made the decisions. Participants also felt that they were not taken seriously. They described feeling ignored and that they lost control of the situation. Some also detailed how they felt robbed of the opportunity to make decisions about their own body—both during labour and in acute situations that resulted in a surgical delivery.

*I kind of felt like I wasn’t allowed to be part of the things that were important to me, and it made me feel like I was watching myself in the room and was just an object that was going to deliver a baby … I felt like I was ignored and excluded from decisions made about my body. I felt I had lost myself*. (w3)

The women likened their loss of control to “*parachuting without the parachute*”, “*going into a burning building*” or a desire to flee the situation by “*jumping out the window*”. One participant described giving birth as if she had lost “*the ground beneath her feet*”. She said she instinctively closed her eyes, tuned out her surroundings and focused on surviving:

*That’s what’s so scary in a way—as long as you have control, you know what’s happening, but when you lose control, you lose the ground beneath your feet and no longer have that foundation, sort of, and instinctively I had to focus on surviving*. (w8)

“I shut it out”: Insufficient time and capacity to process a traumatic birth experience

Everyday life as the mother of an infant was all-consuming and meant that the participants did not have the capacity to process their childbirth experience: “*It was intense and stayed with me after I came home without realising it*” (w1). The women’s coping strategy was to put the trauma behind them. As one woman noted, “*I just shut it out … My anxiety is that I really only—it’s there, but I’m not thinking about it*” (w6). However, a sense of defeat and inability to cope after the traumatic birth overshadowed the joy of becoming a mother. The women struggled to find the emotional availability to feel warmth and love for their child, and felt they lacked the capacity to take an active part in their role as a mother.

*It was really tough, the birth—I was thinking I wasn’t happy about having him, I felt awful … I wasn’t able to take a proper interest in him, I couldn’t muster the energy to take care of him, you know … I’d had such a brutal experience*. (w1)

The participants described feeling inadequate, disappointed in themselves and disheartened. Although the postnatal period was a challenge, as seen in the quotes above, none of the women sought help to process their childbirth experience. Many never talked about the birth, nor the period that followed. One woman noted that, had she been offered counselling shortly after the birth, it would have helped her process the trauma. For those women who did speak to a healthcare professional after the experience, some reported that their experience was downplayed. This led to doubts as to whether their traumatic experience was real or whether they had overreacted. Several of the women pointed to validation, understanding and acknowledgement of their experience as important factors in their ability to process the trauma. One participant described how a lack of validation had tarnished her childbirth experience:

*They didn’t think it was that unusual for the delivery to last that long or for me to lose so much blood … I really didn’t need to hear that. I needed to hear that this was a difficult experience, so that only made it worse*. (w2)

Some participants also expressed a sense of shame and guilt. Several acknowledged that they had waited a long time to get pregnant again, following the traumatic birth, because they did not want to give birth again. Most expressed feelings of ambivalence and considered whether they wanted to go through another pregnancy: “*I was adamant that I wasn’t going to have any more children … Then I got pregnant again. It came as a shock to me. Am I going to have to do this again*?” (w7). One woman explained that her husband was keener to have children than she was, and he had talked her into getting pregnant again. She did not want more children, but at the same time she wanted a sibling for her child: “*I have realized that it’s best to have a sibling … I just shut it out, it’s not up for discussion, yet here we are*” (w6).

They expressed a great need to work through the trauma, and that this realization emerged during the subsequent pregnancy. One woman described it as follows:

*It was actually very surprising—it came over me like a wave of fear—that I had to go through it all again, and I thought, my goodness … I couldn’t see another way out other than having an abortion, quite simply, because I can’t go through it again, and there was a feeling of shame given that I’d got myself pregnant of my own free will*. (w1)

“The baby has to be delivered!”: The new pregnancy triggers a fear of childbirth

Participants described that their previous traumatic birth led to the fear that history would repeat itself, triggering a fear of childbirth. This affected their experience of this new pregnancy and their ideas and feelings about the impending birth. However, they highlighted that there was no escaping the fact that, as one participant stated, “*[t]he baby has to be delivered!*” (w6). Another woman explained,

*It really does something to you. But now I’m pregnant again. Yes, that’s what I think is going to happen this time as well. That you won’t manage to save me. So that’s the biggest fear, I don’t care about pain … But it’s the fear that I’m going to die … That you won’t manage to save me. I’m so scared to go through that experience again … I don’t want to do it again*. (w7)

All the women expressed that their unease or fear about giving birth affected their everyday life. Their fear of childbirth manifested itself in the form of panic, hyperventilation, claustrophobia and stress.

*I then started to really panic about giving birth. So the anxiety I felt about losing her then turned into an anxiety about getting her out, and that sometimes leads to slight claustrophobia, hyperventilating, you know—or I get stressed out*. (w3)

One woman described how she was unable to think about the impending birth or talk to anyone about it. “*After my first conversation with the midwife, I realized that I have to deal with this and that I regretted having to give birth again*” (w5). Others described a sense of fear for the child and for themselves, and some had nightmares. All the women had negative thoughts, to a certain extent. One woman described her thoughts as follows: “*I keep thinking that I’m going to start bleeding on the way to the hospital, and that I’ll go into rapid labour again and that I won’t get there. All these thoughts about not getting there in time*” (w7). Some of the women expressed a fear of dying: “*It’s the fear that I’m going to die*” (w7). Others feared that something would happen to the baby: “*I think about the worst thing that can happen—a stillbirth*” (w3). Some women expressed anxiety in relation to the unpredictability of the outcome of the impending birth: “*But I think the anxiety I’m experiencing now, which I’m struggling with, is because I don’t know what the outcome will be—no one does. That makes me feel very panicky*” (w3).

The participants highlighted that fear of childbirth was a barrier to feeling happy about the pregnancy and preparing for the birth. Several said that they were procrastinating with regards to preparing for the new arrival. One woman reflected on how she did not feel emotionally invested in the pregnancy. She said that she had waited a long time before contacting the midwife and doctor after finding out that she was pregnant—she felt that was going through the motions of what she was supposed to do, but was not emotionally connected. She expressed sadness about this.

*There’s an obstacle in the way that makes me unable … I am looking forward to it, but it’s kind of, I don’t know …. I can’t seem to deal with it. People say things like it will be great to have a new baby in the house … I understand it will happen, but there’s something before that that has to happen and I just can’t face it. That’s the problem*. (w6)

Another woman reflected on how her fear was preventing her from having a good pregnancy. She also expressed a sense of loneliness. She felt alone with her anxiety and wanted to talk to other women in the same situation who understood how she was feeling.

*Yes, it’s meant that I’m not having a very good pregnancy … I’ve been very focused on things like fear … It’s mostly been marked by fear … I’m not doing very well mentally. I feel very alone … No one knows how I feel*. (w7)

### The path to a new childbirth with the hope of mastering fears

The offer of counselling during this new pregnancy had helped several of the women. They felt they were being taken seriously, with one explaining: “*I feel that the hospital is taking me seriously now, and it’s made a huge difference*” (w2). Some of the women had benefitted from writing a birth plan, which gave them a degree of predictability and reassurance. They also expressed the need to talk about their previous traumatic childbirth: “*Being able to have a proper conversation with a midwife about the birth was really helpful*” (w3).

Some of the women were motivated to have a vaginal birth. They were hoping for a good childbirth experience this time, but were unsure how it would go. One of the participants described how a vaginal birth was important for her ability to work through the trauma she had experienced:

*It’s extremely important for me to feel that sense of reassurance so that I can give birth, and it would obviously be best for me to manage it naturally … I need to go through the birth process. If I have a caesarean, I’ll never move on*. (w7)

The women seemed to be able to relate to this sense of ambivalence. For some, however, the fear of a vaginal birth was so heightened that they wanted a caesarean section to ensure predictability and regain a sense of control, despite knowing that this would increase the risk for themselves and their baby:

*I want a caesarean section, and when I voice that, I feel much calmer within myself. Nothing is telling me that it’s wrong, I just know that it feels right, I become calmer, can plan things—it’s more predictable*. (w8)

Another woman also expressed a clear desire to be approved for a planned caesarean and wanted a decision to be made rapidly about this so that she could mentally prepare herself for what would happen. She was unable to relate to a vaginal birth: “*So when they talk about a vaginal birth, I’m just not there, it kind of doesn’t apply to me. I can’t imagine anything other than a caesarean section*” (w6).

It was important for the women to feel seen, heard and included. They described a need for real involvement in the decision making. They did not feel that they had been given enough information to make autonomous choices during the pregnancy and in the preparations for the impending birth: “*The most important thing is just that when they do their job … they remember that I’m there in a way. That I want to be involved and be seen and heard in a way*” (w6).

After previous difficult experiences with healthcare professionals, the women found it difficult to place their trust in them. They highlighted the paradox of being dependent on healthcare professionals in whom they had no confidence: “*You’re supposed to sort of put your trust in the hospital, as it’s the only safety net you have really, but you don’t actually have that*” (w4). However, some of the women did report having confidence in the healthcare professionals and their expertise: “*I generally have a high level of confidence in those with the expertise and think things will go well and that they will take care of me, so I don’t have any concerns in that area*” (w6).

Finally, one woman expressed a hope and desire for more openness around the fear of childbirth.

## Discussion

This phenomenological study investigated the phenomenon of fear of childbirth after a previous traumatic birth. Among the women in our study, the trauma of giving birth strongly impacted their subsequent pregnancy. They struggled to deal with the pregnancy, and the impending birth triggered considerable anxiety. However, they were hopeful that they would master their fears as their pregnancy progressed. In the following sections, we will discuss key findings in the context of relevant research: specifically, a fear of childbirth triggered by a previous traumatic birth; loss of control and the importance of relationships; insufficient capacity to process a traumatic birth experience; life’s vulnerability and predictability; and finally, a hope of mastering their fears in the impending birth.

The first key finding is that the fear of childbirth was closely linked to and could be triggered by a previous traumatic birth experience. The situation that triggered participants’ traumatic memories and fears was an impending birth that was unavoidable. Every day, the women were reminded of their pregnancy and the certainty that they would once again give birth. This sense of being “stuck” was very challenging, and time marched on inexorably towards the situation they feared most. While the birth itself was inevitable, the outcome was unknown, making the fear of childbirth challenging for the women to deal with. This is consistent with other studies that highlight how the fear of childbirth can be a result of a previous negative birth experience (Dencker et al., 2019; Hildingsson, 2015; Sheen & Slade, 2018; Størksen et al., 2012).

The participants’ fear of childbirth stemmed from their fear of obstetric complications, of pain, of losing control, that something would go wrong with them or their baby, or not receiving adequate medical care during labour from the midwife or other healthcare professionals. Similar findings have also been described in other qualitative studies on fear of childbirth (Sheen & Slade, 2018; Slade et al., 2019; Wigert et al., 2020). Our study also shows that fear of childbirth had a significant impact on participants’ everyday lives. They were dogged by negative thoughts and their fear prevented them from enjoying the pregnancy and preparing for the new arrival. They felt a sense of ambivalence that was coupled with the hope that the impending birth would go well. However, there was considerable uncertainty and a real fear of having to go through another traumatic birth. Another study with similar findings shows how women who have experienced a traumatic birth have a strong desire to avoid the same thing happening again (Greenfield et al., 2019).

Pregnancy and childbirth are not only physical events that affect the body—they are also existential events during which women are touched on a deeper level that is connected to life’s vulnerability and unpredictability (Wigert et al., 2020; Yalom, 2011). Childbirth is inherently unpredictable, according to Sheen and Slade (2018). In their study, they point out that fear of childbirth is closely linked to the unpredictable: the fear of not knowing and not being able to plan for the unpredictable. This is supported by findings in our study, where the painful unpredictability associated with childbirth, often expressed as fear of another traumatic birth, pervades the women’s descriptions.

Loss of control was commonly reported by the participants as an important contributing factor to the trauma of the previous delivery. The association between loss of control and perceived trauma has been reported in several studies (Greenfield et al., 2019; Hollander et al., 2017; Nicholls & Ayers, 2007). Perceived control during childbirth can influence whether a delivery is viewed as a positive and satisfying experience, or as a negative experience characterized by fear and trauma (Meyer, 2013; Nicholls & Ayers, 2007). In a concept analysis, Meyer (2013) identified four aspects of control during childbirth: decision making; access to information; personal security, such as trust, respect and support; and physical functioning, in the form of feeling in control of one’s body, emotions and pain. The experiences of the women in our study reflect these aspects. They described a lack of information, autonomy and trust, a loss of agency, and insecurity; some also felt like they were treated like an object.

Participants’ relationship with the midwife in particular, but also with other healthcare professionals, had a significant impact on the their experience of childbirth. They felt safe and that they had a sense of control, to the extent that they experienced the midwife as present and caring during labour, while adequate care involved receiving sufficient information and feeling included in their own birthing process. Unfortunately, the women experienced a lack of these factors in critical moments during labour, which contributed to the traumatic experience. The birth did not unfold as anticipated, and they therefore felt unprepared. Comparable with our findings, a recent Norwegian study found that a negative childbirth experience was associated with inadequate care, disrespect, and exclusion from decision making during labour (Henriksen et al., 2017). Similar results were also found in a recent Spanish study (Rodríguez-Almagro et al., 2019). Birth represents a vulnerable and stressful situation where women may be more dependent on healthcare personnel—and sensitive to inadequate care (Beck, 2015; Brudal, 2000). Stolorow (2015) emphasizes that a difficult and stressful experience can be considered traumatic if it does not take place in a context of emotional understanding—a relational home—where emotional experiences can be shared and understood. This understanding is supported by several other studies that show how the relationship between the expectant mother and the midwife is a key factor in the childbirth experience (Beck, 2015; Dahlberg et al., 2016).

The women felt they were being listened to and were receiving good support from the midwife and doctor during this latest pregnancy, and this made them feel reassured and helped them access their own resources. Beck (2015) emphasizes that it is essential for healthcare professionals to prevent trauma whenever possible. She argues that women need to feel like they are being looked after, treated with respect, and communicated with in a way that makes them feel included in their own birthing process and able to maintain their dignity. The relational support and care women receive before, during and after childbirth could help prevent a traumatic birth experience (Dahlberg et al., 2016; Karlström et al., 2015; Lundgren & Berg, 2007; Lyberg & Severinsson, 2010; Nilsson et al., 2010). Here, the midwives’ and other staff’s treatment of women during the antenatal and postnatal periods, as well as the actual delivery, appears to be the most important factor (Berg & Lundgren, 2004). An awareness of the care that has been provided before, during and after childbirth—and of the importance of women’s subjective birth experience—can give healthcare staff a unique opportunity to improve women’s experience of childbirth and prevent trauma (Ayers et al., 2016) and the corresponding fear of childbirth.

Participants expressed having insufficient capacity to work through their experience in the period after the traumatic birth. They felt overwhelmed and tired, both physically and mentally. The role of mother was all-consuming, and they struggled to be emotionally available for their baby. After the birth, they felt a sense of grief over not being able to cope with either the birth or the transition to motherhood, and several of the women described having lost confidence in healthcare professionals. Participants also expressed a strong need to suppress their traumatic experience, and “shut out” the event in order to move on. This is consistent with the findings of Nilsson and Lundgren (2009), who report that the women in their study blocked out their previous childbirth and avoided everything related to it. According to Gusich (2012), failing to process a traumatic experience, or simply having problems believing and accepting what has happened, are essential characteristics of emotional trauma. This has severe implications for women’s subsequent pregnancies, as it will not be possible to avoid situations that remind them of the traumatic event.

The women in our study described how suppressing their feelings about their new pregnancy was a strategy they employed to avoid dealing with anything reminiscent of their previous childbirth trauma—and the fear that history would repeat itself. This is consistent with previous studies showing that women with a fear of childbirth try to avoid thinking about it and talking about their fears (Beck & Watson, 2010; Eriksson et al., 2006). Indeed, fear of childbirth led participants in our study to procrastinate with regards to preparing for the new arrival, both mentally and practically; however, they nevertheless felt compelled to deal with the impending, unavoidable birth.

Despite a severe fear of childbirth, the women expressed a hope of mastering their fears about the birth. They tried to prepare themselves by reinforcing their inner belief that they would be able to cope with the delivery, whilst recognizing that the birth experience for which they hoped was not guaranteed. Being asked about their expectations for the birth can empower women and help prepare them to deal with the unpredictable (Hildingsson, 2015). Developing a greater tolerance for the unknown is assumed to reduce the fear of childbirth (Sheen & Slade, 2018), and this approach has proven useful in the treatment of women with such a fear (Halvorsen et al., 2010). One of the participants emphasized the importance of having a vaginal birth, as it would enable her to work through her previous traumatic experience of childbirth and master her fears. For some of the women in our study, however, their previous traumatic birth led to a desire for a planned caesarean section, to ensure predictability and regain control.

## Implications for practice

Our findings show that women who have experienced a traumatic birth need to have the opportunity to process the trauma. Routines are needed to identify, support and follow up with women who have experienced a traumatic birth. Future research should focus on how we can best help women work through a traumatic birth experience, with a view towards preventing fear of childbirth. Comprehensive and more adaptable service provision and treatment is needed for women with a fear of childbirth. Future research should therefore also focus on service provision and treatment for this group.

## Conclusion

In this phenomenological study, we explored and discussed women’s experience of fear of childbirth after a traumatic birth. We identified four interconnected constituents: Suboptimal midwifery care, loss of control and agency; insufficient time and capacity to process a traumatic birth experience; “The baby has to be delivered!”, and finally, the path to a new childbirth with the hope of mastering fears. The study sheds light on the association between a previous traumatic birth and a fear of childbirth in expectant mothers. The trauma of giving birth strongly impacted the women’s experiences of their subsequent pregnancy, making it difficult to deal with the new pregnancy and impending birth. Women who experience a traumatic birth need to be given the opportunity to work through the trauma. Routines are thus needed to identify, support and follow up with women who have experienced a traumatic birth. If these women are identified and given the help they need, it could help prevent and alleviate the fear of childbirth in their subsequent pregnancy.
